# Five Commercial Immunoassays for SARS-CoV-2 Antibody Determination and Their Comparison and Correlation with the Virus Neutralization Test

**DOI:** 10.3390/diagnostics11040593

**Published:** 2021-03-25

**Authors:** Václav Šimánek, Ladislav Pecen, Zuzana Krátká, Tomáš Fürst, Hana Řezáčková, Ondřej Topolčan, Karel Fajfrlík, Dalibor Sedláček, Robin Šín, Petr Pazdiora, Hana Zelená, David Slouka, Radek Kučera

**Affiliations:** 1 Department of Immunochemistry Diagnostics, University Hospital in Pilsen, 309 55 Pilsen, Czech Republic; simanek@fnplzen.cz (V.Š.); rezackovah@fnplzen.cz (H.Ř.); topolcan@fnplzen.cz (O.T.); kucerar@fnplzen.cz (R.K.); 2 Laboratory of Immunology, Gennet, 110 00 Prague, Czech Republic; zuzana.kratka@gennet.cz; 3 Faculty of Science, Palacky University in Olomouc, 779 00 Olomouc, Czech Republic; tomas.furst@seznam.cz; 4 Department of Microbiology, University Hospital in Pilsen, 309 55 Pilsen, Czech Republic; Fajfrlik@fnplzen.cz; 5 Department of Infectious Diseases and Travel Medicine, University Hospital in Pilsen, 309 55 Pilsen, Czech Republic; sedlacek@fnplzen.cz (D.S.); sinr@fnplzen.cz (R.Š.); 6 Institute of Epidemiology, Faculty of Medicine in Pilsen, Charles University, 305 99 Pilsen, Czech Republic; pazdiora@fnplzen.cz; 7 Department of Virology, Public Health Institute, 702 00 Ostrava, Czech Republic; hana.zelena@zuova.cz; 8 Department of Biomedical Sciences, Faculty of Medicine, University of Ostrava, 703 00 Ostrava, Czech Republic; 9 Department of Otorhinolaryngology, University Hospital in Pilsen, Faculty of Medicine in Pilsen, Charles University, 305 99 Pilsen, Czech Republic; slouka@fnplzen.cz; 10 Institute of Pharmacology and Toxicology, Faculty of Medicine in Pilsen, Charles University, 323 00 Pilsen, Czech Republic

**Keywords:** antibody, SARS-CoV-2, spike protein, nucleocapsid protein, serological diagnostics, immunoassay, virus neutralization test

## Abstract

There is an ongoing debate as to whether SARS-CoV-2 antibodies can be found in patients who have recovered from COVID-19 disease. Currently, there is no consensus on whether the antibodies, if present, are protective. Our regular measurements of SARS-CoV-2 antibodies, starting in July 2020, have provided us with the opportunity of becoming acquainted with the five different immunoassays. A total of 149 patients were enrolled in our study. We measured the samples using each immunoassay, then performing a virus neutralization test and comparing the results of SARS-CoV-2 antibodies with this test. We observed that the production of neutralizing antibodies is age-dependent. Elderly patients have a higher proportion of high neutralizing titers than young patients. Based on our results, and in combination with the literature findings, we can conclude that the serological SARS-CoV-2 antibody measurement is a helpful tool in the fight against COVID-19. The assays can provide information about the patient’s previous contact with the virus. Anti-spike protein assays correlate well with the virus neutralization test and can be used in the screening of potential convalescent plasma donors.

## 1. Introduction

Severe acute respiratory syndrome coronavirus 2 (SARS-CoV-2) which causes the coronavirus disease 2019 (COVID-19), was first detected in Wuhan, China, in November 2019 [1]. Thus far, the SARS-CoV-2 virus has infected over 100 million individuals with 2.5 million deaths globally and has caused a pandemic which has affected almost every country worldwide [2]. There is an ongoing debate as to whether SARS-CoV-2 antibodies can be found in patients who have recovered from COVID-19 disease. Currently, there is no consensus on whether the antibodies, if present, are protective [3]. Detecting antibodies allows us to confirm that the person was in contact with the virus and developed an immune response against the coronavirus infection. Such a person has a higher likelihood of being protected against a future infection. After the disease, the immune system has a large spectrum of weapons ready for a repeated contact with the virus—especially the specific T cell and humoral immunity [4,5]. Clinical experience gained from studying patients who have been reinfected with other types of coronaviruses indicated that in the 6–12 months following the first infection, the viral load was lower, the period of virus shedding was shorter than in the previous infection, the course of the disease was much weaker, or the disease did not develop [6,7]. 

Test kits for the presence of antibodies against SARS-CoV-2 gradually began to appear on the market in the spring of 2020. Depending on the type of antigen, their sensitivity and specificity varied [8]. Antibodies against SARS-CoV-2 can target several proteins. Currently, nucleocapsid proteins and spike proteins are most commonly used as the targets for serological assays. It is known from other coronaviruses that the spike protein is the main inducer of neutralizing antibodies [9]. Virus neutralizing antibodies protect the organism by blocking the interaction between the virus and the potentially susceptible host cells. 

For a long time, the virus neutralization test (VNT) has been routinely used as a functional test for the assessment of the neutralizing ability of the antibodies produced in the organism [10]. COVID-19 convalescent plasma administration is a type of passive antibody therapy and is used as an experimental treatment option in many countries worldwide [11]. The patients can benefit from the high titer of the virus neutralizing antibodies which can neutralize the virus and help recipients defeat the virus [12].

The aim of this study was to compare five different immunoassays for the determination of SARS-CoV-2 antibodies. First, each assay was compared to the remaining four, and those were then compared to the VNT in order to assess the method’s ability to predict the level of antibody neutralization which could allow the screening of potential plasma donors. We also studied the dependence of the test results on age.

## 2. Material and Methods

### 2.1. Group of Patients

SARS-CoV-2 antibodies were measured between July 24 and August 27, 2020, in 149 patients (55 males, 94 females). All the patients enrolled in the study were subjects from the Pilsen region with a confirmed COVID-19 diagnosis, all with mild clinical symptoms. All diagnostic tests and examinations were performed in the laboratories of University Hospital in Pilsen. The diagnosis was established based on a positive PCR test and/or the presence of clinical symptoms and/or a positive finding using an imaging technique. The blood samples were collected between days 35 and 48 following a positive PCR test result, or the day following a negative PCR result where clinical symptoms were present. It was assumed that by that point the level of IgG antibodies in the patients who were able to produce them would already be detectable. The clinical characteristics of the patient group are shown in Table 1. Informed consent was obtained from all patients enrolled to the study. The study was approved by the Ethics Committee of University Hospital and Medical Faculty in Pilsen on 8 August 2020 (approval number 354/2020).

### 2.2. Serum Samples

Samples of peripheral venous blood were collected using the VACUETTE blood collection system (Greiner Bio-one Company, Kremsmünster, Austria). Serum was separated by 10 minute centrifugation at 1300× *g*, aliquoted into three 500 µL aliquots, and frozen at −80 °C. One aliquot was used for SARS-CoV-2 antibody determination, a second aliquot was used for VNT, and a third aliquot was used as back-up. Aliquots were thawed only once, prior to the immunochemical analyses or VNT.

### 2.3. Immunoassays

Five commercial assays were used in the study. Four of them were chemiluminescent (CLIA) automated assays, and one was an enzyme-linked immunosorbent assay (ELISA) manual assay. We used a CLIA ELECSYS Anti-SARS-CoV-2 assay for the detection of the total-immunoglobulin (total Ig: IgM, IgG, and IgA) antibodies against the nucleocapsid protein (NP) (Roche, Basel, Switzerland); CLIA ARCHITECT SARS-CoV-2 IgG for detection of the IgG antibodies against the nucleocapsid protein (IgG NP) (Abbott, Libertyville, IL, USA); CLIA ACCESS SARS-CoV-2 IgG assay for the detection of the IgG antibodies against the receptor binding domain (RBD) in the S1 subunit of the spike protein (IgG anti-S1-RBD) (Beckman Coulter, Brea, CA, USA); CLIA LIAISON SARS-CoV-2 S2/S2 IgG assay for the detection of the IgG antibodies against the S1 and S2 subunit of the spike protein (IgG anti-S1/S2) (Diasorin, Saluggia, Italy); and an Anti-SARS-CoV-2 ELISA (IgG) assay for the detection of the IgG antibodies against the S1 subunit of the spike protein (IgG anti-S1) (Euroimmun, Lübeck, Germany). All tested assays calculated their results based on the measured signal production in the samples by dividing the stored calibrator result. The overview of the assays used is shown in Table 2. 

### 2.4. Virus Neutralization Test (VNT)

VNTs were performed by the Department of Virology, Public Health Institute in Ostrava, Czech Republic, which is accredited for the performance of this method. 

The VNT was performed using sterile 96-well plates. The SARS-CoV-2 strain was extracted from a clinical sample (hCoV-19/Czech Republic/NRL_9640/2020|EPI_ISL_626593) and CV-1 cells (African green monkey kidney fibroblasts) were used as the susceptible cell line; MEM (minimal essential medium) was buffered with 0.02 mmol/L TRIS-HCl pH 8.1 with the addition of 7.5% BFS (bovine fetal serum) and used as universal diluent. Serum samples were diluted in two-fold dilutions which, after mixing with the virus, resulted in a final serum concentration of 1/10, 1/20 … up to 1/2560. These dilutions were mixed with the virus solution (100 infection doses) and incubated overnight at +4 °C. The next day, 25 µL of CV-1 cell suspension was added into each well. The plates were incubated for further 3–4 days at 37 °C and 5% CO_2_ atmosphere. Then, 25 µL of neutral red dye (1:10,000 aqueous solution) was added into each well and the mixture was incubated for another day at 37 °C and 5% CO_2_. Then, the whole content of the wells was removed, and the uninfected results were red. 

Only live uninfected cells were stained with the neutral red dye, enabling a macroscopic reading, and deeming the use of a microscope unnecessary. The results of the VNT were expressed in the form of virus neutralization titer, which represents an inverted value of the highest dilution of the sample neutralizing the cytopathic effect of the virus for more than 50%. The titer was calculated from the volume of serum and virus at the endpoint of the median tissue culture infectious dose (TCID_50_). Positivity was determined by a titer of 20 and above [13,14].

We compared individual immunoassays to each other. Subsequently, we compared the results of individual immunoassays with VNT. We created a visualization of the comparison of measured concentrations of SARS-CoV-2 antibodies by individual immunoassays, as well as an overview of the data in graphic form. We also assessed the dependence of neutralizing antibody production on age. After correlation with VNT, we suggested two cut-off levels for each immunoassay corresponding to the titers 80 and 160, respectively. We calculated sensitivity (the ability to correctly detect individuals with antibodies) and specificity (the ability to correctly detect individuals without antibodies).

## 3. Statistical Methods

SAS, V. 9.4 (SAS Institute Inc., Cary, NC, USA) and MATLAB, V. R2007b (The MathWorks, Inc., Natick, MA, USA) were used for all statistical analyses. Discrete characteristics are expressed as frequency counts and percentages; continuous characteristics are expressed as means and standard deviations or medians and quartiles, where appropriate. 

The Spearman’s rank correlation was used to identify the best correlating immunoassays. The predictive power of the immunoassays was quantified by the area under the curve (AUC); receiver operating characteristic (ROC) curves were plotted, and cut-off levels, sensitivities and specificities were calculated. The cut-off level was defined as the 95% percentile of the group below a given titer (80 and 160, respectively). These 95% percentiles were calculated by the SAS proc univariate default method and the observation numbered closest to the number of non-missing results multiplied by *p* (where *p* = 0.95 in our case). ROC curves are presented for three of the best correlate assays. 

Graphical statistical displays, histograms, and spaghetti plots are presented. The distribution of the results of a typical immunoassay was highly skewed towards high values; therefore, a linear (Pearson) correlation might be misleading, thus a Spearman’s rank correlation was used. Due to a relatively low number of patients, we advocated for the use of data visualization methods. We used the parallel coordinates plots to visualize the mutual agreement of the five immunoassays and their correspondence with the VNT results. Age dependence of measured parameters was also analyzed. This relationship can also be non-linear. Therefore, a scatter plot graph was first displayed, and then correlation by the Spearman’s correlation coefficient was calculated if the relationship to age was monotonous.

## 4. Results

**Figure 1:**
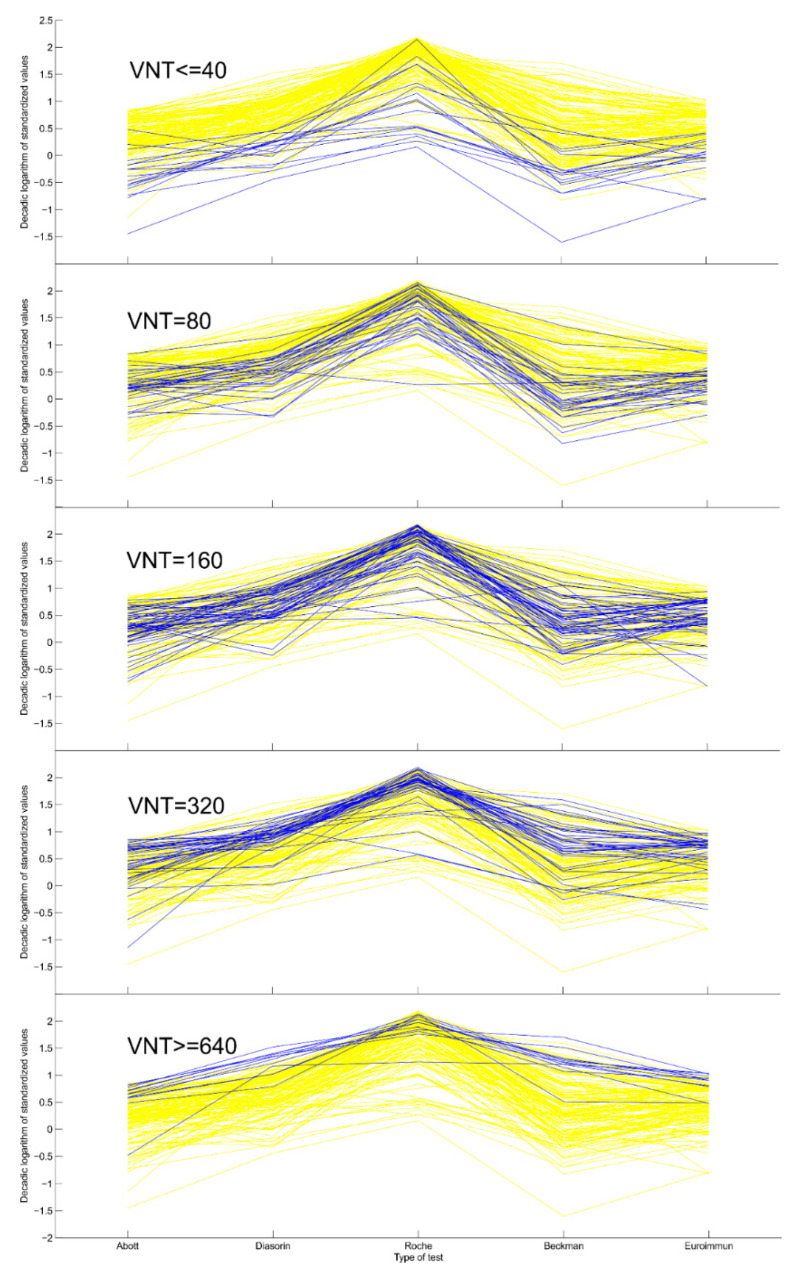
Visualization of the comparison of measured concentrations of SARS-CoV-2 antibodies by individual assays and overview of the data. Legends: The results of each test were standardized by taking the (decadic) logarithm of the ratio of the test result to the cut-off for negativity (see Table 4 and the text for more details). Each yellow line represents a single sample. In each panel, samples of particular VNT value are highlighted blue. We may observe the overall agreement of the five tests (Table 3) and the general correspondence of the VNT results with the five tests.

The summary of the results of individual immunoassays used in the study is shown in Table 3. The values of medians depend on the design of individual assays. The highest median was achieved by Roche, following by Diasorin. The distribution of the results in individual assays determines the distance of the points from the *x*-axis in the visualization of the comparison of measured concentrations of SARS-CoV-2 antibodies (Figure 1).

The correlation between all immunoassays used in the study and the correlation of individual immunoassays with VNT are shown in Table 4. Assays targeted against spike protein (Diasorin, Beckman Coulter and Euroimmun) showed a correlation with each other. The correlation between the other two assays (Abbott and Roche) was weaker due to the different construction of the assays (detection of antibodies against the N protein). The correlation of individual immunoassays with the VNT reveals that assays targeted against the spike protein (Diasorin, Beckman Coulter and Euroimmun) showed correlation with the VNT, and assays targeted against the nucleocapsid protein (Abbott and Roche) exhibited less correlation. The closest correlation was demonstrated between VNT and the Diasorin assay.

Figure 1 represents the visualization of the comparison of measured concentrations of SARS-CoV-2 antibodies by individual assays and provides an overview of the data. The *x*-axis shows the individual methods for the determination of antibodies (Abbott, Diasorin, Roche, Beckman, Euroimmun). The *y*-axis plots the decimal logarithms of the standardized antibody values. Each test has a cut-off value below which the result is considered negative (1.4 for Abott, 12 for Diasorin, 1.0 for Roche, 0.8 for Beckman, and 0.8 for Euroimmun; see Table 1). Each measured value was divided by this threshold and then logarithmized. For example, the result of Diasorin test, 120, is thus transformed into *log(120/12) = 1*. This transformation ensures the comparability of individual methods with each other on the one hand (we convert everything to a multiple of the negativity limit) and, on the other, allows for a more even distribution of data points on the *y*-axis. Each yellow line in the graph connects values measured from the same serum sample by different methods. In the individual panels, only samples with the appropriate VNT antibody titer are marked in blue. In subgroup ≤40, all samples with the lowest titers 20 and 40 are marked in blue together; in subgroups 80, 160 and 320, the titers 80, 160 and 320 are plotted in blue; in subgroup ≥640, all samples where the highest titers are 640 and 1280 are marked in blue together. Samples with low titers of neutralizing antibodies are located at the bottom of the yellow “field”; a low titer of neutralizing antibodies corresponds to a low value of antibodies in all methods used. With increasing the VNT titer, blue lines appear at higher levels, until the highest titers are used at the top of the yellow field. In the case of VNT ≤ 40, one expects the blue lines to lie at the bottom of the yellow region. This happens very consistently in cases of Diasorin and Euroimmun, but it does not happen so consistently in the case of Roche.

### 4.1. Dependence of Neutralizing Antibody Titers on Age

**Figure 2:**
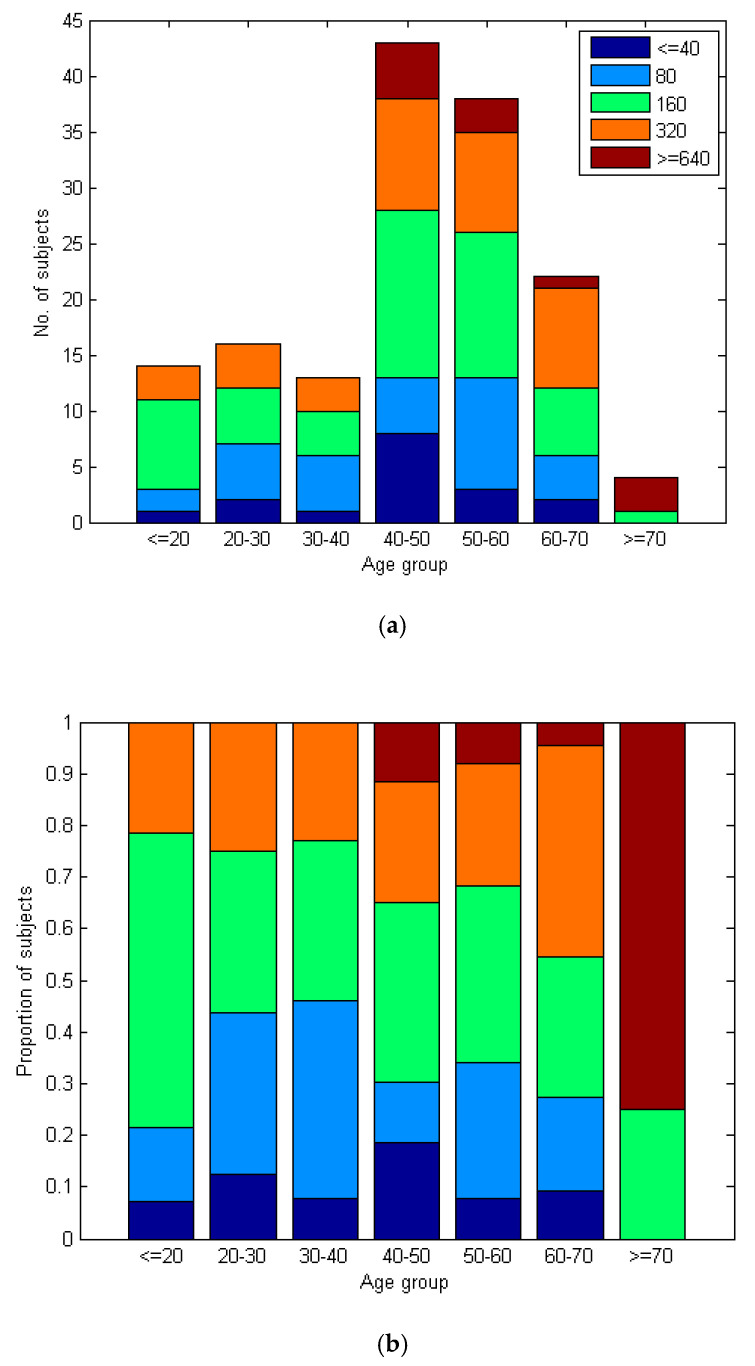
(**a**) The number of VNT results across several age groups. (**b**) The proportion of VNT results across several age groups. Legend: The proportion of VNT ≥ 320 subjects visibly grows with age, while the proportion of VNT ≤ 80 decreases.

The values of neutralizing antibody titers in the VNT test correlate significantly with the age of patients. The correlation between age and the result of the VNT test is low (c = 0.17), but marginally significant (*p* = 0.04). Figure 2 shows that elderly patients have a higher proportion of people with high VNT titers than young people. The percentage of higher titers (≥320) increases continuously with age, as seen in Figure 2b.

### 4.2. Cut-Off Values for Screening Suitable Donors of Convalescent Plasma

**Figure 3:**
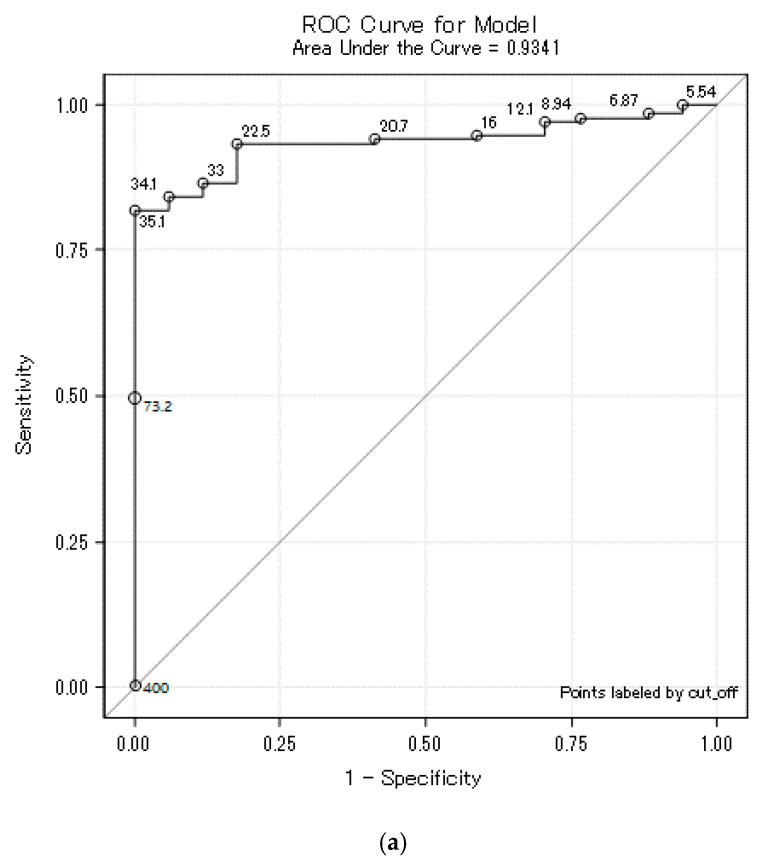

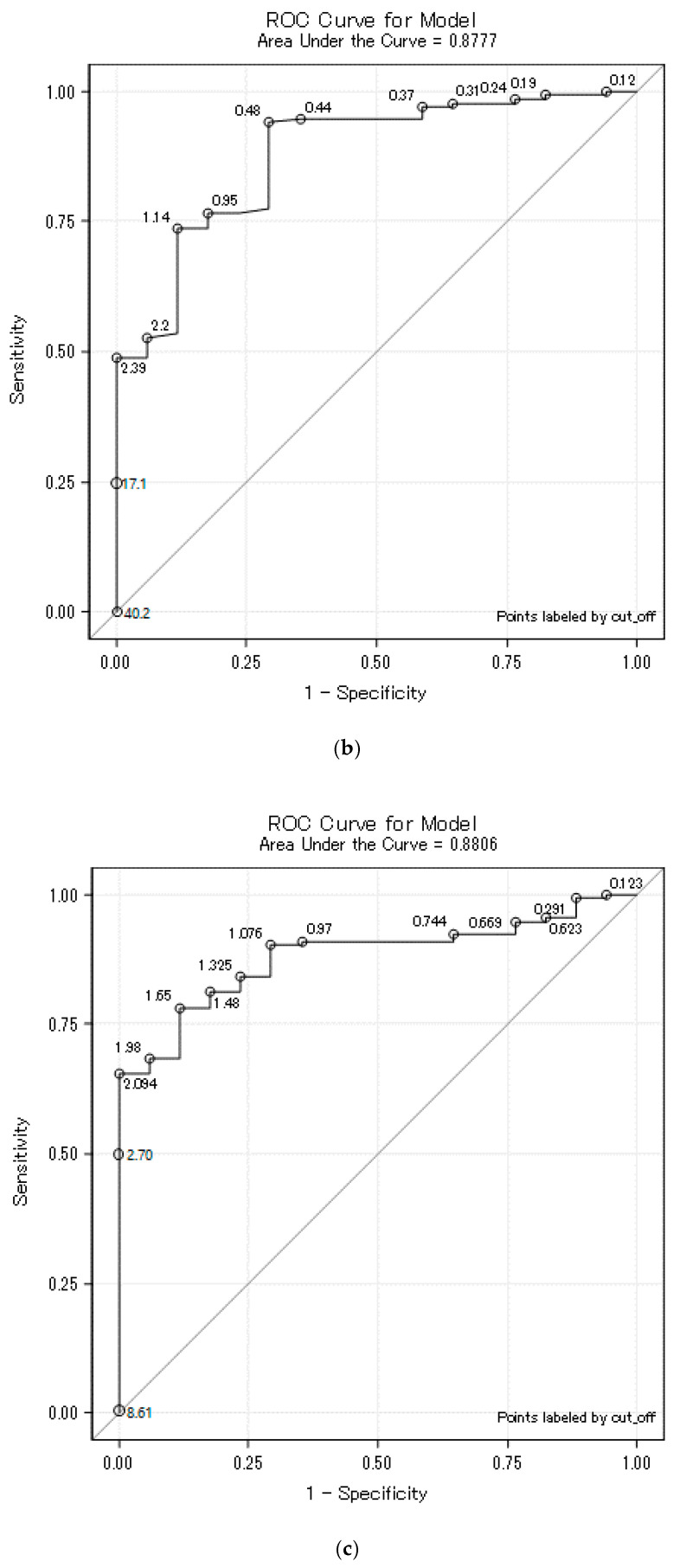
(**a**) Diasorin cut-off for titer 80. (**b**) Beckman Coulter cut-off for titer 80. (**c**) Euroimmun cut-off for titer 80. Legend: ROC curve for Diasorin, Beckman Coulter and Euroimmun, titer <80 vs. ≥80 where the ROC curve is labeled by all possible cut-offs. These ROC curves are atypical, and, above all, their vertical parts increase at the point of 100% specificity, demonstrating that it was not targeted at 100% specificity, but 95%. For some methodologies, however, it accidentally led to 100% specificity. For cut-off values for individual methods, see Table 4.

**Figure 4:**
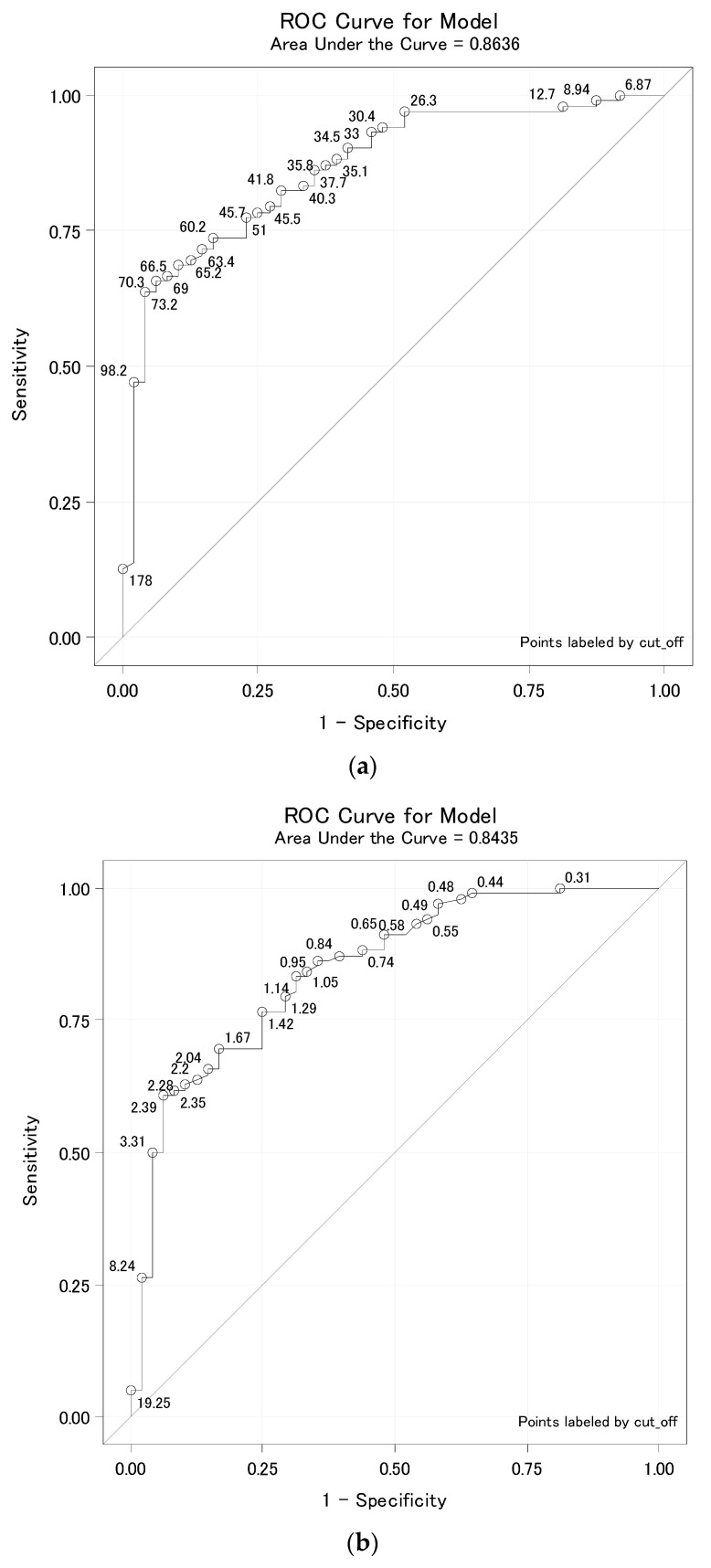

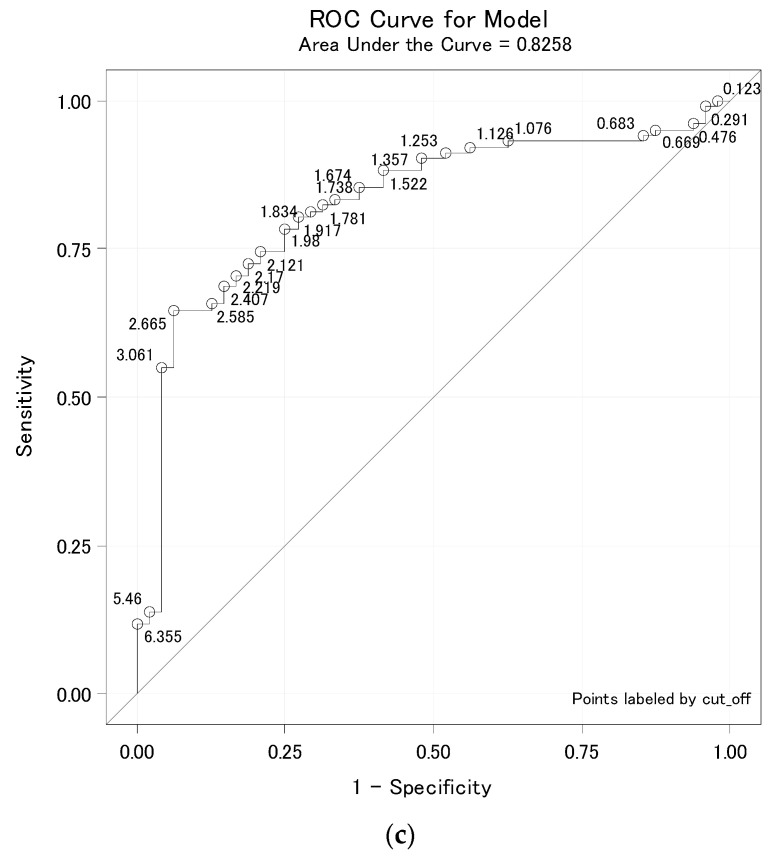
(**a**) Diasorin cut-off for titer 160. (**b**) Beckman Coulter cut-off for titer 160. (**c**) Euroimmun cut-off for titer 160. Legend: ROC curve for Diasorin, Beckman Coulter and Euroimmun, titer <160 vs. ≥160 where the ROC curve is labeled by all possible cut-offs. These ROC curves are already typical. Thus, specificity can be targeted as close to 95%. For cut-off values for individual methods, see Table 4.

For the purpose of screening suitable convalescent plasma donors, we calculated cut-off values for individual immunochemical assays (Table 5). Based on these cut-off values of IgG antibodies measured in patients, it is possible to predict the values of neutralizing antibodies. The IgG antibody values measured by the Diasorin, Beckman Coulter, and Euroimmun assays correlated best with the VNT result (Table 4). Although the cut-off was defined as the 95% percentile of the group below a given titer, for some tests this led to 100% specificity at titer 80. The ROC curves for titer 80 have an atypical course and the 100% specificity was achieved randomly, which can be explained from the ROC curves (Figure 3a–c and Figure 4a–c). At a false positive of 0%, and a specificity of 100%, the curves increased for many cut-offs. For Roche and Abbot assays, their low correlation with the VTN caused lower sensitivities, especially at 160.

## 5. Discussion

Serological tests represent an important tool in our fight against the COVID-19 pandemic. Serological assays were very quickly developed, and in a short time their use gave rise to routine diagnostics capable of determining a patient’s previous contact or infection with the SARS-CoV-2 virus [15]. When we started routinely measuring SARS-CoV-2 antibodies in July 2020, we were presented with the opportunity to test and become acquainted with the five different immunoassays. Based on their biological differences, we can recognize two groups of immunoassays [16]: assays targeted against the spike protein, and assays targeted against the nucleocapsid protein. During the first wave of the pandemic, we primarily wanted to find out who had been in contact with the virus. For this purpose, the Roche assay seemed to be the best choice because of its construct. The concept of the total antibody measurement meant the highest sensitivity, which is visible in Figure 1 when the group with titer ≤40 was evaluated. The blue line which connects the values of the same samples in all the assays is most visible at the background of all (yellow) samples in the Roche assay. This finding is in concordance with the findings of other authors [17,18,19]. 

Experience with other strains of coronaviruses shows that the spike protein is the target for neutralizing antibodies. The nucleocapsid protein is shielded by a viral membrane, and antibodies targeted against the nucleocapsid protein cannot directly neutralize the virus [20]. Neutralizing antibodies protect the host by binding to specific proteins on the SARS-CoV-2 virus to neutralize its ability to bind to the host cellular receptor angiotensin-converting enzyme 2 (ACE2), and after the fusion of viral and cellular membranes, enter into the host cell. Antibodies also facilitate the recognition and killing of the virus by phagocytic immune cells [21]. 

It was on the basis of this that COVID-19 convalescent plasma administration started in many hospitals as the experimental treatment option; as passive antibody therapy [11]. According to the current recommendation, the convalescent plasma should have at least a 160 titer of the neutralizing antibodies, although titer 80 is still acceptable as a minimum threshold value [22]. To assess the ability of tested assays to predict the presence of neutralizing antibodies, we performed VNT and compared the results of titers with the values of SARS-CoV-2 antibodies measured by individual assays. In agreement with other studies [23,24,25,26], we have proved a very good correlation of anti-spike protein assays with the VNT. The blue lines in anti-spike protein assays are visible in Figure 1 (Diasorin, Beckman Coulter and Euroimmun) in individual subgroups (starting with the titer ≤40 to the final subgroup titer ≥640); they pass more evenly through the diagram from the bottom to the top, which is a sign of the individual assays correlating better with the VNT. Correlation coefficients are presented in Table 3. To assure the titer of the neutralizing antibodies at 80 or 160, respectively, we suggested a cut-off level and calculated sensitivity and specificity. We also plotted ROC curves and calculated AUC values for each assay (Table 4). It can be seen from Table 4 that the assays targeted against the nucleocapsid protein mainly provide information about the contact with the virus. In our evaluation, these assays achieved very low sensitivity, especially for titer 160. Therefore, ROC curves are presented only for anti-spike protein assays (Figure 3a–c and Figure 4a–c), potential predictors of the values of neutralizing antibodies. Cut-off levels for Euroimmun and Diasorin can be compared with the available study by Valdivia et al. [26]. 

Valdivia et al. worked with a group of 51 patients, which is a smaller group than ours, and the patients were hospitalized, which means that the course of the diseases was probably worse than in our group of patients. However, at the current level of knowledge, it is hard to hypothesize the affect that this difference could have had on the results of the serology tests obtained in both studies. They targeted a neutralization titer of 160 and 95% specificity. They achieved higher cut-off levels for Euroimmun (8.9) compared to our cut-off value (3.06). However, for Diasorin assay, the cut-offs were closer together: the cut-off by Valdivia et al. was 90.6 AU/mL, compared to our cut-off 73.2 AU/mL. 

Based on these findings, we can conclude that the cut-off values of anti-spike protein assays shown in Table 4 can be added to the general rules for searching for convalescent plasma donors (patients with the confirmed COVID-19 infection who have recovered). Adding the cut-off values of anti-spike protein assays from Table 4 as additional criteria to the screening of the suitable donors of the convalescent plasma will ensure a high probability of titers of neutralizing antibodies.

The last issue we wanted to address is the correlation of the production of neutralizing antibodies with age. Such information is still rare, and some authors point out the necessity of obtaining such data [27]. We tried to create a homogeneous group of patients with respect to the course of the disease, so that the levels of antibodies were comparable across the age spectrum. The patients enrolled in the study were not hospitalized, all with mild clinical symptoms. The blood samples were taken at the same time period from the onset of the disease. Spearman’s rank correlation was used, which cannot be influenced by outliers. In accordance with another Czech study [13], we proved a positive correlation of the titer of neutralizing antibodies with the increasing age of patients (Figure 2). 

## 6. Conclusions

Serological assays that measure SARS-CoV-2 antibodies are helpful tools in the COVID-19 pandemic situation. The assays can provide information about the patient’s contact with the virus. Anti-spike protein assays correlate well with the VNT and can be used to screen potential convalescent plasma donors. In our group of patients, we proved a positive correlation between the titer of neutralizing antibodies and older age groups.

## Figures and Tables

**Table 1:** Clinical characteristics of the patient group.

Characteristic	Category	*n* (%)
**Gender**	Female	94 (63)
	Male	55 (37)
	Total	149 (100)
**Age**	Median [range]	47.5 (4–73)
**PCR**	positivity/negativity	138 (92)/11 (8)
**Clinical symptoms**	fever ≥ 37.5 °C	69 (46)
	dry cough	51 (34)
	difficulty breathing or shortness of breath	33 (22)
	loss of taste or smell	59 (39)
	headache	68 (45)
	tiredness	105 (70)
**Risk factors**	obesity	17 (11)
	smoking (last 10 years)	17 (11)
	diabetes	12 (8)
	cardiovascular disease	13 (9)
**Flu vaccination**	for season 2019	9 (6)

**Table 2:** Immunoassays of SARS-CoV-2 antibodies used in the study.

Manufacturer	Methodology	Antigen Used	Evaluation of the Results			Manufacturer’s Catalog Number
			Negative	Gray Zone	Positive	
Abbott	CLIA	IgG NP	<1.4	-	≥1.4	6R86
Diasorin	CLIA	IgG anti-S1/S2	<12	12–15	≥15	311450
Roche	CLIA	Total Ig NP	<1.0	-	≥1.0	09 203 095 190
Beckman Coulter	CLIA	IgG anti-S1 RBD	<0.8	0.8–1.0	≥1.0	C58961
Euroimmun	ELISA	IgG anti-S1	<0.8	0.8–1.1	≥1.1	EI 2606-9601 G

Legend: The individual assays report the results as follows: Abbott as an index value (S/C); Diasorin as arbitrary units per mililiter (AU/mL); Roche as cut-off index (COI); Beckman Coulter as an index value (S/CO); Euroimmun as a ratio (number only).

**Table 3:** Summary of the results of individual immunoassays used.

Manufacturer	*n*	Median	Minimum	Maximum	Lower Quartile	Upper Quartile
Abbott	149	2.66	0.10	10.12	1.51	4.91
Diasorin	149	66.50	5.54	400	34.5	110
Roche	149	73.45	1.84	155	31.8	107
Beckman Coulter	149	1.88	0.12	40.2	0.72	5.47
Euroimmun	149	2.46	0.12	8.61	2.46	4.54

**Table 4:** Correlation of the individual immunoassays for the determination of SARS-CoV-2 antibodies and their correlation with the virus neutralization test (VNT).

	Abbott	Diasorin	Roche	Beckman	Euroimmun	VNT
Abott	1.00	0.49	0.65	0.65	0.49	0.49
Diasorin		1.00	0.41	0.76	0.84	0.72
Roche			1.00	0.39	0.38	0.38
Beckman Coulter				1.00	0.73	0.68
Euroimmun					1.00	0.63
VNT						1.00

Legends: All the (Spearman’s) correlation coefficients are significant at *p* < 0.001. The diagonal is highlighted in yellow, correlations above 0.7 are marked in red, and correlations below 0.5 are marked in blue.

**Table 5:** Cut-off value with sensitivity and specificity for VNT titer 80 and 160.

Manufacturer	Titer 80				Titer 160			
	Cut-Off	Sensitivity	Specificity	AUC	Cut-Off	Sensitivity	Specificity	AUC
Diasorin	35.1	82%	100%	0.9341	73.2	64%	96%	0.8636
Beckman Coulter	2.39	49%	100%	0.8777	3.31	50%	96%	0.8435
Euroimmun	2.09	65%	100%	0.8806	3.06	55%	96%	0.8258
Abbott	4.26	35%	100%	0.8819	6.00	26%	96%	0.7168
Roche	68.4	59%	94%	0.8514	131.4	13%	96%	0.7111

Legend: The individual assays report the results as follows: Abbott as an index value (S/C); Diasorin as arbitrary units per milliliter (AU/mL); Roche as cut-off index (COI); Beckman Coulter as an index value (S/CO); Euroimmun as a ratio (number only); Cut-off is defined as the 95% percentile of the group below the titer 80 and 160; AUC, area under the curve.

## Data Availability

Data are available by the corresponding author.
